# Acute tissue damage induced by monopolar microelectrodes and radiofrequency in vocal cords after transoral cordectomy

**DOI:** 10.1016/j.bjorl.2019.11.006

**Published:** 2019-12-12

**Authors:** Jorge Basterra, Nezly Mosquera, Natsuki Oishi, Ana Pérez, Enrique Zapater

**Affiliations:** a Hospital General Universitari de València, ENT Department, Valencia, Spain; b University of Valancia, Faculty of Medicine, Valencia, Spain; c Hospital General Universitari de València, Pathology Department, Valencia, Spain

**Keywords:** Microelectrodes, Vocal cord, Tissue damage, Radiofrequency

## Abstract

**Introduction:**

In 2006 and 2009, we reported the levels of acute and chronic tissue damage after cordectomy associated with use of the microlectrodes using high frequency energy. In 2010, we shifted to radiofrequency rather than high frequency electrogenerators.

**Objective:**

The aim of this study is to evaluate acute tissue damage in the larynx after cordectomy using microelectrodes coupled to a radiofrequencygenerator.

**Methods:**

We studied 22 patients with a stage T1 glottic squamous cell carcinoma. The patients were randomly assigned to the two operating mode: cutting or coagulation (11 patients each mode). The strength of the study is that there are no previous studies on the effect of radiofrequency in human vocal cord.

**Results:**

Tissue damage was milder when microelectrodes were coupled to a 4 MHz generator operating in the cutting mode. Thus, when using microelectrodes and radiofrequency, we recommend that the cutting mode be used for epithelial incision and the coagulation mode to treat the stroma and muscle and for final hemostasis.

**Conclusion:**

Microelectrodes and radiofrequency in transoral laryngeal surgery produced mild tissue damage and offer an excellent alternative to the use of high frequency energy.

## Introduction

In 2006 and 2009, we reported the levels of acute and chronic tissue damage after cordectomy associated with use of the CO_2_ laser and microlectrodes (ME) using high frequency (HF) energy and found that the extent of tissue damage was similar regardless of the methods used.^1^, ^2^

The vocal fold healing process is unique and differs from that in other body locations characterized by a replacement of collagen type 1 by type 3 collagen.^3^ We measured the extent of acute tissue damage caused by the surgery because it increases the risk for scar formation and poor functional voice outcome.

In 2010, we shifted to radiofrequency (RF) rather than HF electrogenerators. The reason for this change is based on the fact that the tissue resistance to RF radiation is lower than that to HF radiation. When RF radiation is delivered, the cell walls are bridged via a capacitive effect, and the energy is released directly into the cells, minimising the energy requirement^4^ and consequently the injury to the surrounding normal tissues.

The aim of this study is to evaluate acute tissue damage in larynx after cordectomy using MEs coupled to a RF generator.

## Methods

We studied 22 patients (17 males and 5 females) with a stage T1 glottic squamous cell carcinoma.^5^ Oncological results have been published elsewhere.^6^, ^7^ All cordectomies^8^ were performed via direct suspension laryngoscopy using MEs designed in 2006 by the first author^6^ coupled to a 4 MHz RF generator (Sutter Medizintechnik Freiburg, Germany) delivering 15 W. The patients were randomly assigned to the two operating modes: cutting or coagulation (11 patients each mode).

**Figure 1:**
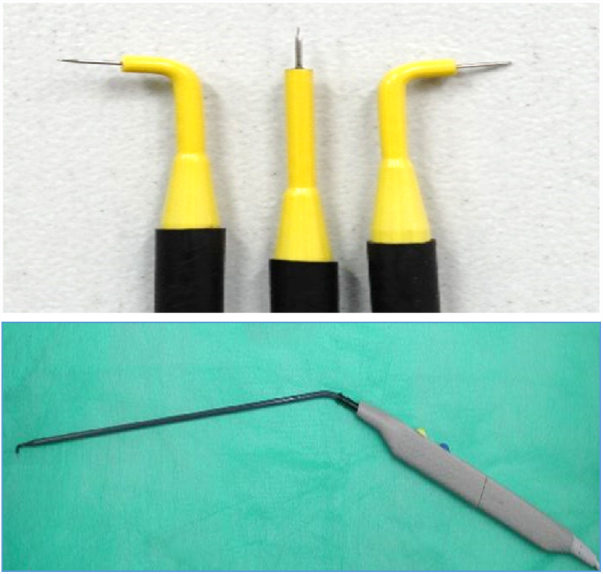
Monopolar microelectrodes. Metal tips are 3 mm × 0.3 mm. Lengths’ of the shaft is 21 cm. Microelectrodes attached to the hand piece.

**Figure 2:**
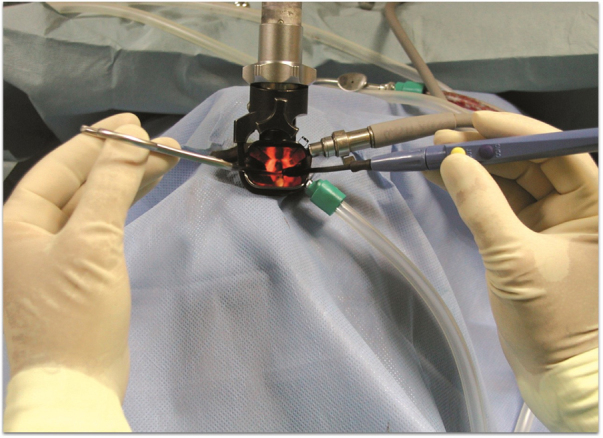
Bivalve laryngoscope in place. The angled fórceps (left hand) and the angled microelectrode (right hand), hands are off the field. Surgical microscope is then used.

Other surgical instruments were: conventional microsurgery forceps, aspiration tube and laryngoscopes for anterior commisure and occasionally a bivalve laryngoscope as used for CO_2_ laser surgery^7^ (Figure 1, Figure 2).

The variables analysed were age, gender, cutting/coagulation mode, epithelial tissue damage status, and the extent of stromal damage.

Surgical specimens for this study (2–4 mm in diameter) were taken from the surgical berth after removing the tumor. Microsurgery forceps were used for this specimen, and then immersed in formalin solution and stained with hematoxylin and eosin which is the best method to quantify acute tissue damage.^1^

The histopathological study considered:

Epithelial damage at each incision site: we evaluated the presence of deformed pyknotic nuclei, thrombotic blood vessels and lymphatics, and cauterisation artefact zones.

Stromal damage: a millimeter ruler was used to measure the thickness of specimens and the extent of damage to cord muscle fibres. The morphological parameter employed to assess tissue damage was the thickness of denatured collagen fibres.

Each sample field was divided into three equal portions and damage was graded as follows: a) No sclerosis: the changed area did not exceed 1/3 of the field or was less than 0.6 mm in width; b) Mild sclerosis: the changed area covered 1/3 to 2/3 of the field and was 0.6–1.3 mm in width; c) Moderate sclerosis: the changed area exceeded 2/3 of the field and was 1.3–1.7 mm in width; d) Severe sclerosis: the whole field was changed.

A single pathologist performed all histological evaluations in a blinded manner.

All patients were informed about the methodology and goals of the study, and signed consent forms. The study design was approved by our institutional review board, the approval number is 25042013. Patient confidentiality was protected.

## Results

The mean patient age was 62.4 years (SD = 13.1 years): 82 % were male and 18 % were female.

**Figure 3:**
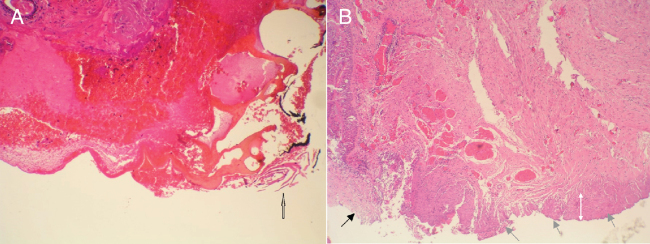
A, Specimen of the surgical berth associated with the use of microelectrodes and radiofrequency in the coagulation mode (H&E, 40×). The epithelium exhibits a coagulation artefact (white arrow), necrosis, dehiscence, and pyknotic nuclei (empty arrow); sub epithelial haemorrhage is also apparent. B, Specimen of the surgical berth associated with the use of microelectrodes and radiofrequency in the cutting mode (H&E, 20×). The black arrow indicates the area of incision; the epithelium is preserved. The gray arrows indicate the deep margin corresponding to the cut zone. The white arrows indicate a small area with mild stromal damage. The change in coloration and thickening of the collagen bands covers less than one-third of the field.

Epithelial damage: All patients operated upon using the coagulation mode showed epithelial damage. The cutting mode did not cause significative tissue damage (Fig. 3).

Stromal damage: In those operated upon using cutting mode, the extent of stromal tissue damage was minimal (no sclerosis) in 72.7 % of patients: the width of the area exhibiting morphological changes was < 0.6 mm; 7.3 % of patients exhibited slight or moderate damage (the width of the damaged area was up to 1.3 mm). No patient exhibited moderate or severe damage. When the coagulation mode was used, the extent of stromal tissue damage was mild in 54.2 % of patients, moderate in 27.3 %, and nil in 18.2 % (no sclerosis). No patient exhibited severe damage.

We found no significant association between gender or age and the extent of tissue damage (*p* = 0.87 and 0.09, respectively). However, younger patients tended (*p* = 0.09) to show less tissue damage.

## Discussion

The strength of the study is that there are no previous studies on the effect of RF in human vocal cord.

Several studies have evaluated histological changes in the upper aerodigestive tract after cold scalpel surgery, use of a CO_2_ laser, and dissection using ME and a HF electrogenerator. All studies defined collagen denaturation as the key element of damage. Therefore, we measured the thickness of the denatured collagen zone to quantify acute tissue damage produced by dissection using ME and RF of the human vocal cord.

Several histopathological studies of various modes of RF applied at different sites have appeared, and have revealed the following:

RF deep tissue reduction: RF induces ion agitation within tissues, increasing the local temperature and creating thermal lesions in the deep submucosal stroma without damaging the surface; also fibrosis develops during healing, leading to reductions in tissue volume. This technique has been used to treat benign pathologies such as hypertrophied turbinates, obstructive tonsillar hypertrophy, the soft palate and tongue base hypertrophy.

Several histopathological studies of the pharynx after tissue reduction using RF surgery have been published. Plzak analysed 10 palatine tonsils specimens subjected to RF thermotherapy: histologically, the submucosa showed no sign of increased fibrosis reflecting scarring. The architecture of the lymphoid germinal centres was normal, as was the extent and type of vascularisation.^9^

RF ablation: it has been used to treat benign laryngeal pathologies, such as recurrent papillomatosis, large laryngeal cysts and stenosis (Basterra, unpublished data), and to perform posterior cordotomy.^10^ When using a CO_2_ laser to treat the nasal mucosa, several authors have observed reduced numbers of (less active) seromucinous glands, fibrosis of the connective tissue stroma, and long-term impairment of mucociliary transport.^11^ In comparison, no apparent functional loss was evident after RF tissue ablation.

### Clinical applicability of the study

The extent of vocal cord tissue damage after transoral cordectomy using ME and an RF generator (4 MHz) delivering 15 W was generally mild in both the epithelium and stroma. The reason for this finding may be that RF energy is conducted through the cell membranes to be absorbed within target cells, thus not propagating to adjacent normal tissue.

**Figure 4:**
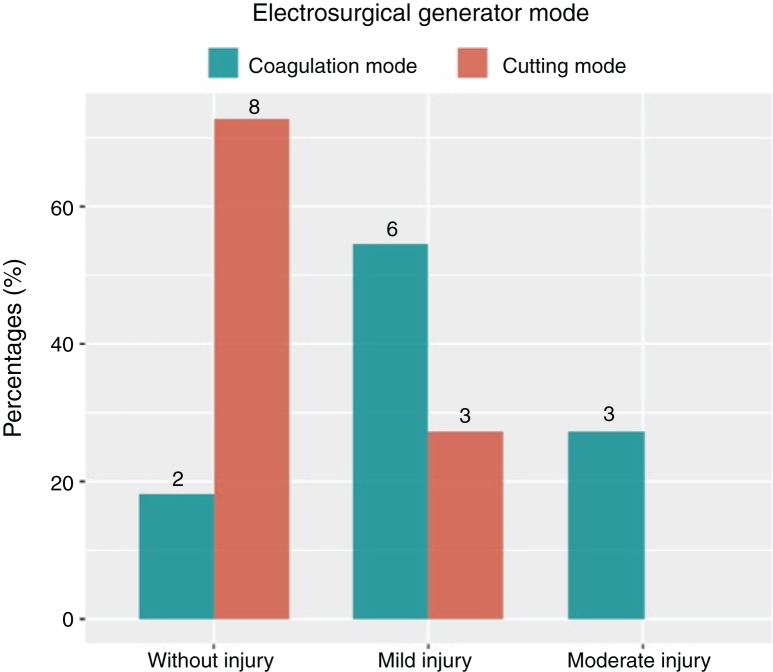
Tissue damage scores of the generator modes used. The cutting mode causes less damage than the coagulation mode.

Tissue damage was milder when ME were coupled to a 4 MHz generator operating in the cutting mode (Fig. 4). Thus, when using ME (Fig. 4) and RF, we recommend that the cutting mode be used for epithelial incision and the coagulation mode to treat the stroma and muscle, and for final haemostasis. To minimize the tissue damage is relevant in larynx where the functional outcome, in addition to the oncological result, improves the quality of life.

## Conclusion

ME and RF in transoral laryngeal surgery produced mild tissue damage and offer an excellent alternative to the use of high frequency energy.

## Conflicts of interest

The authors declare no conflicts of interest.
